# Are Dating App Algorithms Making Men Lonely and Does This Present a Public Health Concern?

**DOI:** 10.2196/70594

**Published:** 2025-04-07

**Authors:** Eric Balki

**Affiliations:** 1 Faculty of Health and Medicine Department of Health Lancaster University Lancaster United Kingdom

**Keywords:** dating apps, mental health, men, algorithm, anxiety, depression, loneliness

## Abstract

During the pandemic, dating apps emerged as essential platforms connecting users amid social isolation, experiencing rapid growth in engagement and profile creation. This paper examines the evolution of these apps, highlighting their shift from facilitating offline encounters to promoting match accumulation for revenue. In particular, the study investigates gender disparities, addictive behaviors, and algorithmic match throttling that disproportionately impact men’s psychological well-being. Drawing on evidence linking dating app use to increased depression and anxiety, the analysis calls for regulatory intervention to eliminate pay-for-advantage models and ensure fair, healthy user experiences, thereby mitigating adverse public health outcomes.

This paper deals with issues revolving around heterosexual dating, which currently forms the largest marketplace in dating apps [1]. During the pandemic with frequent lockdowns, dating apps provided an opportunity for people to connect [2]. In a short span of time, there was a surge in dating profiles on these apps. Just like Peloton, the home exercise machine company that found a way of appealing to users to use stationary bikes and treadmills, dating apps attracted a vast range of young adults looking for a connection or relationship, at a time when isolation and loneliness was high [3].

At the start of the pandemic, the way the apps were working was quite simple. When two users accepted each other’s profile, they had a match. Browsing through and evaluating other user’s profiles was the central activity, eventually leading to an offline encounter where possible [4]. Dating apps, however, slowly started to not facilitate offline encounters but rather feed into the user’s desire of accumulating matches [5].

Even before the pandemic, more than half of the users on dating apps reported not going on in-person dates, and this percentage was especially higher for women [6], with them looking for validation, gratification, and a boost in self-worth instead [7]. It is very likely this has worsened even further, with a large majority of users not going on in-person, offline dates [8].

Even those who are now in relationships find it hard to quit these apps—keeping them for gratification purposes [9], especially in women who accumulate matches to satisfy the need for belongingness or as a replacement for relational or sexual intimacy, getting value from social acceptance and approval [10].

In the last two years, the problem has exacerbated significantly. Women report being inundated with so many matches that it is hard from them to make a decision on choosing someone to engage with properly [11]. At the same time, men are on the opposite end of the spectrum, where they get very few responses and must purchase expensive, paid features and subscriptions, which allow them to get a limited amount of priority over nonpaying members [12].

Men continue to form the largest group of paid subscribers to dating apps [12]. Dating apps are focused on maximizing profit activities, especially as many of them are now owned by publicly listed companies [13]. Losing a subscriber means losing revenue, so it is conjectured that dating apps are motivated to keep their paying customers as long as they possibly can, as a successful offline encounter mediated by the dating app would lead to the loss of that subscriber [12]. Unlike other social media platforms, there is no upside in successful encounters from a revenue-generating perspective. Men are also more likely to quit a dating app if they have found someone in person [12].

Dating apps are like casinos in a way, in that they have to strategize where the reward needs to be—just enough to keep users coming back for more, but the reward cannot be so high that users walk away and not return, which can increase addictive behavior [14]. They have gamified the process of meeting a partner, invoking “gambler” tendencies, thus leading to behaviors that are addictive or even compulsive [15]. Now, sophisticated artificial intelligence (AI) tools are being deployed by dating apps to maximize subscriber revenue, which is potentially causing excessive harm to the psychological profiles of a large number of men and hindering the development of healthy relationships, which could become a large public health concern [16]. Thus, there is both algorithmic and natural throttling of the mutual acceptance matching process on these apps, which is driving an increasing frustration in men as well as impacting their female users [16].

This is the fallacy in the approach by dating app companies, as the pool of paid subscribers will eventually, and inevitably, fall as the frustration leads users to find alternative ways of finding dates.

It has become extremely important for researchers to study the psychological traits and well-being of users of dating apps [17], especially with the changes and advances in algorithms and use of AI. As things stand, there are very few studies that have looked at dating app use; the algorithms being deployed by such companies; match throttling; and the associated psychological outcomes, especially in men.

There is a need for studies that move away from simply associating correlations between dating app use and psychological outcomes, including mere use associated with decreased well-being. Authors need to distinguish which specific activities, features, or experiences may be leading to undesired effects. While it may not always be possible to understand how the backend of the apps works or what algorithms may be at play, real-time testing with users could reveal basic issues revolving around authentic matching [18]. The design of such studies will have to be carefully considered to ensure the cross-sectional design does not end up ruling out self-selection, that is, attracting lonely individuals and those in fear of being single [19].

The majority of the population are reliant on dating apps to meet prospective partner, and such apps are having an impact on their mental health; these factors may very well make this into a public health concern. The Centers for Disease Control and Prevention’s [20] Household Pulse Survey estimates that depression and anxiety are now affecting 34.2% of the population, making them the two most common mental health disorders in the United States, and to some extent, the increased numbers have been associated with dating app use [21].

Some studies have linked dating apps directly with higher depression and anxiety, especially the frequency and length of use [17]. Symptoms from these disorders can further impact social skills, making the formation of healthy relationships even more difficult, thus leading to a downward spiral in the populace [18].

This is an urgent problem that needs regulatory action from the Western governments, where dating app algorithms should provide fair conditions to all users. Match throttling and pay-for-advantage models should be disallowed as they have a disproportionately negative impact on the health of young men.

If such action is not taken, then over time we will have a population of young, productive men who grow tired of using dating apps to find a partner and come out feeling lonely, depressed, and anxious, thus impacting in other areas of their life and their ability to be productive. Therefore, rather than helping people find partners, dating apps may be failing the very users they rely upon for their income.

## Abbreviations

AI: artificial intelligence
